# Some results on the multipartite Ramsey numbers *m*_*j*_(*C*_3_,*C*_*m*_,*n*_1_*K*_2_,*n*_2_*K*_2_,…,*n*_*i*_*K*_2_)

**DOI:** 10.1016/j.heliyon.2022.e11431

**Published:** 2022-11-03

**Authors:** Yaser Rowshan, Mostafa Gholami, Stanford Shateyi

**Affiliations:** a Department of Mathematics, Institute for Advanced Studies in Basic Sciences (IASBS), Zanjan 66731-45137, Iran; b Department of Mathematics and Applied Mathematics, School of Mathematical and Natural Sciences, University of Venda, P. Bag X5050, Thohoyandou 0950, South Africa

**Keywords:** Ramsey numbers, Multipartite Ramsey numbers, Stripes, Cycle

## Abstract

The graph $K_{j\times t}$ is a graph which is complete and multipartite which includes *j* partite sets and *t* vertices in each partite set. The multipartite Ramsey number (M-R-number) $m_{j}(G_{1},G_{2},\ldots ,G_{n})$ is the smallest integer *t* for the mentioned graphs $G_{1},G_{2},\ldots ,G_{n}$, in a way which for each *n*-edge-coloring $\left(G^{1},G^{2},\ldots ,G^{n}\right)$ of the edges of $K_{j\times t}$, $G^{i}$ contains a monochromatic copy of $G_{i}$ for at least one *i*. The size of M-R-number $m_{j}(nK_{2},C_{7})$ for j≥2, n≤6, the M-R-number $m_{j}(nK_{2},C_{7})$ for j=2,3,4, n≥2, the M-R-number $m_{j}(nK_{2},C_{7})$ for each j≥5, n≥2, the M-R-number $m_{j}(C_{3},C_{3},n_{1}K_{2},n_{2}K_{2},\ldots ,n_{i}K_{2})$ for j≤6, and $i,n_{i}\geq 1$, and the size of M-R-number $m_{j}(C_{3},C_{3},nK_{2})$ for j≥2 and n≥1 have been calculated in various articles hitherto. We acquire some bounds of M-R-number $m_{j}(C_{3},C_{3},n_{1}K_{2},n_{2}K_{2},\ldots ,n_{i}K_{2})$ in this essay in which i,j≥2, and $n_{i}\geq 1$, also the size of M-R-number $m_{4}(C_{3},C_{4},nK_{2})$ for each n≥1 is computed in this paper.

## 1 Introduction

After the publication of article [1] by Erdös and Rado in 1956, Ramsey theory has attracted the attention of many mathematicians due to its many applications in various fields like graph theory, geometry, logic, and number theory [2]. The classical Ramsey number for the given numbers $n_{1},\ldots ,n_{k}$, is the smallest integer *n* in a way that there is some 1≤i≤k for each *k*-coloring of the edges of complete graph $K_{n}$, such that there is a complete subgraph of size $n_{i}$ whose edges are all the *i*th color.

However, instead of complete graphs, one can consider any other class of graphs and their subgraphs. One can refer to [3], [4], [5], and their references for further studies.

Assume that $K_{j\times t}$ is a complete and multipartite graph which contains *j* parts and each part contains *t* vertices. As for the graphs $G_{1},G_{2},\ldots ,G_{n}$, the multipartite Ramsey number (M-R-number) $m_{j}(G_{1},G_{2},\ldots ,G_{n})$ is the lowest integer *t* (if there exist) so that for each *n*-coloring of the edges of $K_{j\times t}$ for instance $\left(G^{1},G^{2},\ldots ,G^{n}\right)$, $G^{i}$ contains a copy of $G_{i}$ which is monochromatic for at least one *i*.

Burgeet et al. have done a research on the value of $m_{j}(G,H)$, under the condition which *G* and *H* are both complete multipartite graphs in [6]. Recently, the exact value of $m_{j}(G,H)$ was calculated for certain graph classes, see [7], [8], [9] alongside related references, which normally can be boosted to various colors, see [10], [11], [12], [13], [14], [15], [16], [17]. The multipartite Ramsey number $m_{j}(K_{1,m},G)$ in case that *G* is considered as a path or a cycle including *n* vertices and j=2,3, has been studied in [14]. In [18], authors have studied the value of $m_{j}(nK_{2},C_{n})$, in case that j≥2 and n∈{3,4,5,6}. The exact values of $m_{j}(nK_{2},C_{7})$, in case that n≥2 and j≤4 have been obtained in [19], and the values of $m_{j}(nK_{2},C_{7})$, in case that j≥5 and n≥1 have been obtained in [20].

In the current essay, we provide a number of bounds on M-R-number $m_{j}(C_{3},C_{3},n_{1}K_{2},n_{2}K_{2},\ldots ,n_{i}K_{2})$, where i,j≥2, $n_{i}\geq 1$, and $m_{4}(C_{3},C_{4},nK_{2})$ for each n≥1. Specifically, we prove two bellow-mentioned outcomes:

Theorem *For any positive integers* $i,j,n_{i}$ *, where* $i\geq 2,n_{i}\geq 1$ *,*

• $1+\lfloor \frac{\sum_{k=1}^{k=i}(n_{k}-1)}{j-5}\rfloor \leq m_{j}(C_{3},C_{3},n_{1}K_{2},n_{2}K_{2},\ldots ,n_{i}K_{2})$ *, where* 6≤j≤10 *.*
• $m_{j}(C_{3},C_{3},n_{1}K_{2},n_{2}K_{2},\ldots ,n_{i}K_{2})\leq \lfloor \frac{2\sum_{k=1}^{k=i}(n_{k}-1)}{j-5}\rfloor +1$ *, where* j≥6 *.*

Theorem *Suppose that n is a positive integer, then:*

$$m_{4}(C_{3},C_{4},nK_{2})=\left\{ \begin{array}{cc} \lceil \frac{2n}{3}\rceil ;\; & n=3k+2,\;k\geq 1,\\ \lceil \frac{2n}{3}\rceil +1;\; & otherwise. \end{array}\right.$$

All considered graphs *G* are simple, finite, and undirected. Take G=(V,E) as a graph, the order of *G* is the cardinality of its vertex set. That is, the order of *G* is |V(G)|. An *n* stripe (also called Matching of size *n* or $nK_{2}$) of a graph *G* is defined as a complex of *n* edges, not having a common vertex. A maximum matching is a matching of maximum magnitude among all matchings in the graph. The degree of *v* is denoted by $\deg_{G}(v)$ for a vertex v∈V(G), and the neighbors of *v* is denoted by $N_{G}(v)$. The neighborhood of a vertex $v\in V(G)\cap X_{j}$ is defined by $N_{X_{j}}(v)=\{u\in V(X_{j})|uv\in E(G)\}$. A graph $G^{\prime }=(V^{\prime },E^{\prime })$ is a subgraph of one other graph G=(V,E) if and only if $V^{\prime }\subseteq V$ and $E^{\prime }\subseteq E$. $C_{n}$ denotes a cycle with *n* vertices. The complex of edges between partite sets $X_{i}$ and $X_{j}$ is denoted by $\left[X_{i},X_{j}\right]$. If S⊆V(G) is any subset of vertices of *G*, then the subgraph of *G* induced by *S* (G[S]) will be the graph that has *S* as set of vertices for it and contains all the edges of *G* that have both endpoints in *S*. Graph $\bar{G}$ is the complement of a graph *G* on the same set of vertices as for *G* in a way which between two vertices (u,v) in $\bar{G}$, there will be an edge if and only if there is no edge in between (u,v) in *G*. For two graphs *G* and *H*, the join of *G* and *H* is a graph made from disjoint copies of *G* and *H* by connecting each vertex of *G* to each vertex of *H* and denoted by G⊕H. The union of two simple graphs $G_{1}=(V_{1},E_{1})$ and $G_{2}=(V_{2},E_{2})$ is the simple graph with vertex set $V_{1}\cup V_{2}$ and edge set $E_{1}\cup E_{2}$. The union of $G_{1}$ and $G_{2}$ is indicated by $G_{1}\cup G_{2}$. An isomorphism from a simple graph *G* to a simple graph *H* is a bijection f:V(G)→V(H) in a way that uv∈E(G) if and only if f(u)f(v)∈E(H). We contemplate “*G* is isomorphic to *H*”, written G≅H, in case there is an isomorphism from *G* to *H*. For convenience, suppose that [n]={1,2,…,n}. *G* is *n*-colorable to $\left(G_{1},G_{2},\ldots ,G_{n}\right)$ if *G* contains an *n*-edge decomposition like $\left(G^{1},G^{2},\ldots ,G^{n}\right)$, for which $G_{i}⊈G^{i}$ for each i=1,2,…,n.

## 2 M-R-number $m_{j}(C_{3},C_{3},n_{1}K_{2},\ldots ,n_{i}K_{2})$

In current section, we provide some bounds for $m_{j}(C_{3},C_{3},n_{1}K_{2},n_{2}K,\ldots ,vn_{i}K_{2})$. We commence with Theorem 2.1.

The significant simple instance is the value of $R(C_{3},C_{3})$. It declares that in a party including many people, either three of them know each other or three of them do not know each other. The calculation of this Ramsey number has been questioned in mathematical competitions such as Putnam.

Theorem 2.1 *[3]**,*$R(C_{3},C_{3})=6$*.*

Definition 2.2 [21] Take $K=K_{j\times t}$ as a complete multipartite graph with partite sets $X_{i}$, where for each i∈[j], $|X_{i}|=t$. Let *G* be a subgraph of *K*. $G^{⁎}\subseteq K$ is defined in a way that its vertex sets is $V(G^{⁎})=\{v_{1},v_{2},\ldots ,v_{m}\}$, where 1≤m≤j, $v_{i}=X_{i}$ for each *i*, and two vertices $v_{i}$ and $v_{j}$ are adjacent in $G^{⁎}$ in case each vertex of $X_{i}$ is adjacent to all vertices of $X_{j}$ in *G*.

For each $5\geq j,n_{i}\geq 1$, the value of $m_{j}(C_{3},C_{3},n_{1}K_{2},n_{2}K_{2},\ldots ,n_{i}K_{2})$, was obtained in the below-mentioned theorem.

Theorem 2.3 *[21]* *For each of the positive integers* $j,i,n_{i}$ *, which* 2≤j≤5 *, and* $i,n_{i}\geq 1$ *,*

$$m_{j}(C_{3},C_{3},n_{1}K_{2},n_{2}K,\ldots ,n_{i}K_{2})=\infty .$$

Proof Let *t* be an arbitrary integer. It will be proved that $K=K_{j\times t}$ is (i+2)-colorable to $\left(C_{3},C_{3},n_{1}K_{2},n_{2}K_{2},\ldots ,n_{i}K_{2}\right)$. Consider $\left(G^{1},G^{2},\ldots ,G^{i+2}\right)$ as an (i+2)-edge-coloring of $K_{j\times t}$, in which for each r≥3, $G^{r}$ is a null graph, that means $G^{1}\cup G^{2}≅K$. As j≤5, by Theorem 2.1 and Definition 2.2, *K* can be decomposed into two disjoint $G_{1}^{⁎}$ and $G_{2}^{⁎}$, so that $G_{i}^{⁎}$ is $C_{3}$-free (for example if j=5, then $G_{1}^{⁎}=G_{2}^{⁎}=C_{5}^{⁎}$), which means *K* is 2-colorable to $\left(C_{3},C_{3}\right)$. Now, since *K* is 2-colorable to $\left(C_{3},C_{3}\right)$, $G^{1}\cup G^{2}≅K$, for each r≥3 we have $G^{r}$ is a null graph, and *t* is an arbitrary integer, so:

$$m_{j}(C_{3},C_{3},n_{1}K_{2},n_{2}K_{2},\ldots ,n_{i}K_{2})=\infty .$$

 □

Suppose that *i* and $n_{1},n_{2},\ldots ,n_{i}$ are i+1 positive integers, the following theorem gives the exact value of $m_{6}(C_{3},C_{3},n_{1}K_{2},n_{2}K_{2},\ldots ,n_{i}K_{2})$, for each $i,n_{i}\geq 1$.

Theorem 2.4 *[21]**Related to any positive integers*$i,n_{i}\geq 1$*,*

• $m_{6}(C_{3},C_{3},nK_{2})=n$*.*
• $m_{6}(C_{3},C_{3},n_{1}K_{2},n_{2}K_{2},\ldots ,n_{i}K_{2})=1+\sum_{j=1}^{j=i}(n_{j}-1)$*.*

Suppose that j≥6 is a positive integer, in the next theorem we get both lower and upper bounds related to M-R-number $m_{j}(C_{3},C_{3},n_{1}K_{2},n_{2}K_{2},\ldots ,n_{i}K_{2})$, for each $i\geq 2,n_{i}\geq 1$.

Theorem 2.5 *Related to any positive integers*$i,j,n_{i}$*, where*$i\geq 2,n_{i}\geq 1$*,*

*(I):* $1+\lfloor \frac{\sum_{k=1}^{k=i}(n_{k}-1)}{j-5}\rfloor \leq m_{j}(C_{3},C_{3},n_{1}K_{2},n_{2}K_{2},\ldots ,n_{i}K_{2})$*, where*6≤j≤10*.*
*(II):* $m_{j}(C_{3},C_{3},n_{1}K_{2},n_{2}K_{2},\ldots ,n_{i}K_{2})\leq \lfloor \frac{2\sum_{k=1}^{k=i}(n_{k}-1)}{j-5}\rfloor +1$*, where*6≤j*.*

Proof By Theorem 2.4, (I) and (II) holds for j=6.

**Proof I**: Assume that j≥7 and j=5+r, where r∈{2,3,4,5}. Now, consider $K=K_{j\times t}$ which has *j* parts $X_{i}=\{x_{1}^{i},x_{2}^{i},\ldots ,x_{t}^{i}\}$ for i=1,2,…,j and $t=\lfloor \frac{\sum_{k=1}^{k=i}(n_{k}-1)}{j-5}\rfloor$.

Consider $\left(G^{1},G^{2}\right)$ as a 2-edge-coloring of *K*, where $G^{1}≅K_{5\times t}\cup K_{r\times t}$, and $\bar{G^{1}}=G^{2}$ in which $K_{5\times t}≅G[X_{1},\ldots ,X_{5}]$, and $K_{r\times t}≅G[X_{6},\ldots ,X_{5+r}]$. It is straightforward to check if $G^{2}$ is a bipartite graph with 5×t vertices in one part and r×t vertices in other part. That is $G^{2}≅K_{m_{1},m_{2}}$, where $m_{1}=5\times t$, and $m_{2}=r\times t$. By definition $G^{1}$, one can check that $G^{1}\subseteq 2K_{5\times t}$. Therefore, since $G^{1}\subseteq 2K_{5\times t}$, we can decompose the edges of $G^{1}$ into two classes, say $G_{1}^{1}$, $G_{2}^{1}$, where $G_{i}^{1}≅C_{5}^{⁎}\cup H_{i}$, so that $H_{1}\cup H_{2}=K_{r\times t}$, and $H_{i}$ is $C_{3}$ free. Therefore, $G_{i}^{1}$ is $C_{3}$-free for i=1,2, that is $G^{1}$ is 2-colorable to $\left(C_{3},C_{3}\right)$.

Now, consider $G^{2}$, we need to show that $G^{2}$ is *i*-colorable to $\left(n_{1}K_{2},n_{2}K_{2},\ldots ,n_{i}K_{2}\right)$. As $t=\lfloor \frac{\sum_{k=1}^{k=i}(n_{k}-1)}{j-5}\rfloor$ and $G^{2}≅K_{m_{1},m_{2}}$, where $m_{1}=5\times t$, and $m_{2}=r\times t$, so $m_{2}$ have at most $\sum_{k=1}^{k=i}(n_{k}-1)$ vertices. Hence, one can decompose the edges of $G^{2}$ into *i* classes, say $G_{1}^{2},G_{2}^{2},\ldots ,G_{i}^{2}$, where $G_{r}^{2}$ is a bipartite graph with $n_{r}-1$ vertices in one part and $m_{1}$ vertices in other part. In other words $G_{r}^{2}≅K_{m_{1},n_{r}-1}$ for each r∈[i]. So $|M_{r}|\leq n_{r}-1$ for each r∈[i], where $M_{r}$ is a maximum matching (M-M) in $G_{r}^{2}$, that is $G_{r}^{2}$ is $n_{r}K_{2}$-free. Which means that $G^{2}$ is *i*-colorable to $\left(n_{1}K_{2},n_{2}K_{2},\ldots ,n_{i}K_{2}\right)$.

Therefore, since $G^{1}$ is 2-colorable to $\left(C_{3},C_{3}\right)$ and $G^{2}$ is *i*-colorable to $\left(n_{1}K_{2},n_{2}K_{2},\ldots ,n_{i}K_{2}\right)$, we have $K=G^{1}\cup G^{2}$ is (i+2)-colorable to $\left(C_{3},C_{3},n_{1}K_{2},n_{2}K_{2},\ldots ,n_{i}K_{2}\right)$. Hence, for each 6≤j≤10, and $i\geq 2,n_{i}\geq 1$, we have $m_{j}(C_{3},C_{3},n_{1}K_{2},n_{2}K_{2},\ldots ,n_{i}K_{2})\geq t+1$, where $t=\lfloor \frac{\sum_{k=1}^{k=i}(n_{k}-1)}{j-5}\rfloor$.

**Proof II**: Perceive $K=K_{j\times t}$ with partite sets $X_{i}=\{x_{1}^{i},x_{2}^{i},\ldots ,x_{t}^{i}\}$ for each i∈[j], and $t=1+\lfloor \frac{2\sum_{k=1}^{k=i}(n_{k}-1)}{j-5}\rfloor$. Consider $\left(G^{1},G^{2},\ldots ,G^{i+2}\right)$ as an (i+2)-edge-coloring of *K*, where $n_{k}K_{2}⊈G^{k+2}$ for each 1≤k≤i. As $|M_{k}|\leq n_{k}-1$ for each k∈[i], where $M_{k}$ is a M-M in $G^{2+k}$, set $M=\cup_{k=1}^{k=i}M_{k}$, hence $\left|M|\leq \sum_{k=1}^{k=i}(n_{k}-1\right)$. Now, since j≥7, and i≥2, the following claim is issued by us:

Claim 2.6 *For each*$\left(j_{1},j_{2},\ldots ,j_{p-5}\right)$*, where*$j_{i}\in [j]$*, at least one vertex x of*$X_{j_{1}}\cup X_{j_{2}}\cup \ldots \cup X_{j_{p-5}}$*, exists, such that*x∉V(M)*.*

Proof By contradiction, suppose that there exists at least one $\left(j_{1},j_{2},\ldots ,j_{p-5}\right)$, so that $X_{j_{1}}\cup X_{j_{2}}\cup \ldots \cup X_{j_{p-5}}\subseteq V(M)$. Now, since $|X_{j}|=t$, $X_{j_{1}}\cup X_{j_{2}}\cup \ldots \cup X_{j_{p-5}}\subseteq V(M)$ and $t=1+\lfloor \frac{2\sum_{k=1}^{k=i}(n_{k}-1)}{p-5}\rfloor$, we have $|M|\geq 2\sum_{k=1}^{k=i}(n_{k}-1)+1$. Hence, by the pigeon-hole principle there exists at least one *k*, 1≤k≤i, so that $|M_{k}|\geq n_{k}$, which is a contradiction. □

Therefore, since j≥7, by Claim 2.6, there are at least 6 parts of *K*, say $X_{1},X_{2},\ldots ,X_{6}$, so that at least one vertex $x_{r}$ of $X_{r}$ exists, such that $x_{r}\notin V(M)$, for each r∈[6]. Without loss of generality (W.l.g) we may assume that $x_{r}=x_{1}^{r}$ for each r∈[6]. Therefore, $K_{6}≅G[\{x_{1}^{1},\ldots ,x_{6}^{1}\}]\subseteq G^{1}\cup G^{2}$. So by Theorem 2.1, either $C_{3}\subseteq G^{2}$ or $C_{3}\subseteq G^{1}$. Hence, $m_{j}(C_{3},C_{3},n_{1}K_{2},n_{2}K_{2},\ldots ,n_{i}K_{2})\leq t+1$ and complete is the proof. □

As R(3,3)=6, it can be checked that $m_{6}(C_{3},C_{3},K_{2},\ldots ,K_{2})=1$. Assume that $\left(G_{1},G_{2},G_{3},G_{4}\right)$ is a 4-edge decomposition of $K_{7}$, where $G_{1}≅C_{5}$, $G_{2}≅C_{5}$, $G_{3}≅K_{1,6}$, and $G_{4}≅K_{1,5}$. Hence it is clear that $K_{7}≅G_{1}\cup G_{2}\cup G_{3}\cup G_{4}$, $C_{3}⊈G_{i}$ for i=1,2, and $2K_{2}⊈G_{i}$ for i=3,4. Which means, $m_{7}(C_{3},C_{3},2K_{2},2K_{2})\geq 2$. Also, it is easy to say that $m_{7}(C_{3},C_{3},2K_{2},2K_{2})=2$. So the bound (*I*) is sharp.

## 3 M-R-number $m_{j}(C_{3},C_{4},nK_{2})$

In this segment, we obtain a lower bound on M-R-number $m_{j}(C_{3},C_{4},nK_{2})$. Also, we provide a formula of this M-R-number for j=4 and each n≥1. As a bipartite graph has no odd cycle, we have $m_{2}(C_{3},C_{4},nK_{2})=\infty$. Now we begin with Theorem 3.1, Theorem 3.2

Theorem 3.1 *[22]* *Given any complete multipartite graph* $K=K_{n_{1},\ldots ,n_{m}}$ *, where* $n_{1}\leq n_{2}\leq \ldots \leq n_{m}$ *, and* $n_{m}\leq n_{1}+n_{2}+\ldots +n_{m-1}-1$ *, then* $M=\lfloor \frac{N}{2}\rfloor$ *, in which* $N=n_{1}+n_{2}+\ldots +n_{m}$ *and M is a M-M in K.*

Theorem 3.2 [21], [23]*For each positive integer*n≥2*,*

• $m_{4}(C_{3},C_{4})=2$*.*
• $m_{3}(C_{3},C_{4})=3$*.*
• $m_{3}(C_{3},C_{4},nK_{2})=n+2$*.*

For computing the exact value of the size of $m_{j}(C_{3},C_{4},nK_{2})$, we need some lemmas as follows:

Lemma 3.3 *Let*$K_{n,m,2}$*be a complete 3-partite graph with*n,m≥1*, then*$K_{n,m,2}$*is* 2*-colorable to* $\left(C_{3},C_{4}\right)$*.*

Proof Consider $K=K_{n,m,2}$ with partite sets $X_{1}=\{x_{1}^{1},x_{2}^{1},\ldots ,x_{n}^{1}\}$, $X_{2}=\{x_{1}^{2},x_{2}^{2},\ldots ,x_{m}^{2}\}$, and $X_{3}=\{v,v^{\prime }\}$. Consider $\left(G^{1},G^{2}\right)$ as a 2-edge-coloring of *K*, where $G^{2}≅K_{1,n}\cup K_{1,m}≅G[\{v\},X_{1}]\cup G[\{v^{\prime }\},X_{2}]$, and $G^{1}≅\bar{G^{1}}$. By definition $G^{i}$, it is easy to say that $G^{1}$ is bipartite, so $C_{3}⊈G^{1}$ and it is clear that $C_{4}⊈G^{2}$, which means that $K_{n,m,2}$ is 2-colorable to $\left(C_{3},C_{4}\right)$. □

**Figure 1:**
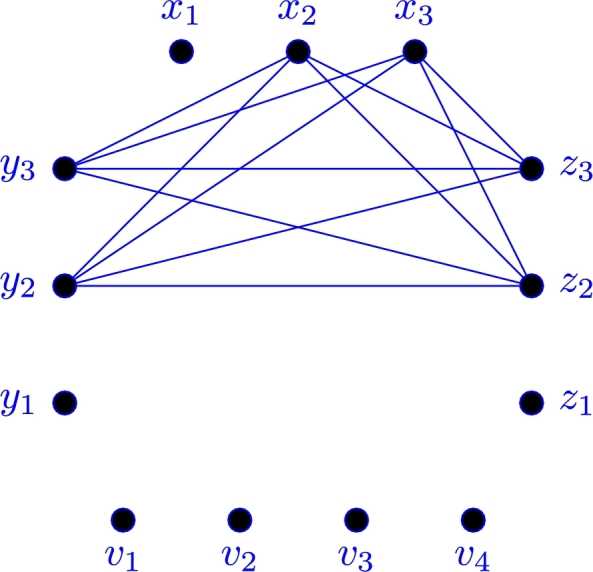
$\bar{H}≅K_{2,2,2}$.

Lemma 3.4 *Suppose that H is a subgraph of* $K_{3,3,3,4}$ *, where* $\bar{H}$ *is shown in* Fig. 1 *. For any red-blue coloring of the edges of H say* $\left(G^{r},G^{b}\right)$ *, either* $C_{3}\subseteq G^{r}$ *or* $C_{4}\subseteq G^{b}$ *.*

Proof Let X,Y,Z,V are the partition sets of V(H), where $X=\{x_{1},x_{2},x_{3}\},Y=\{y_{1},y_{2},y_{3}\},Z=\{z_{1},z_{2},z_{3}\}$, and $V=\{v_{1},\ldots ,v_{4}\}$. Therefore $H≅4K_{1}⊕(K_{3,3,3}\setminus K_{2,2,2})$. By contrary, suppose that $C_{3}⊈G^{r}$ and $C_{4}⊈G^{b}$. Therefore, as $C_{3}⊈G^{r}$, by considering $K=K_{3}≅G[\{x_{1},y_{1},z_{1}\}]$, there is at very least one edge of E(K) in $E(G^{b})$. Now by considering $E(K)\cap E(G^{b})$ we expect the below-mentioned three cases:

**Case 1**: $|E(K)\cap E(G^{b})|=3$, that is $K\subseteq G^{b}$. Since $C_{4}⊈G^{b}$, at least one edge exists between $\left\{x_{1},y_{1}\right\}$ and $\left\{z_{2},z_{3}\right\}$ in $G^{r}$. W.l.g suppose that $x_{1}z_{2}\in E(G^{r})$. By symmetry there exists at least one edge between $\left\{z_{1},y_{1}\right\}$ and $\left\{x_{2},x_{3}\right\}$ in $G^{r}$. W.l.g suppose that $y_{1}x_{2}\in E(G^{r})$. Also, one can say that $|N_{G^{r}}(z_{3})\cap \{x_{1},y_{1}\}|\geq 1$, otherwise $C_{4}\subseteq G^{b}[V(K)\cup \{z_{3}\}]$, a contradiction. W.l.g let $x_{1}z_{3}\in E(G^{r})$. So, if $|N_{G^{r}}(x_{1})\cap V|\geq 2$, then as $C_{3}⊈G^{r}$, we have $C_{4}\subseteq G^{b}[\{z_{2},z_{3}\},V]$, a contradiction again. Therefore $|N_{G^{r}}(x_{1})\cap V|\leq 1$, that is $|N_{G^{b}}(x_{1})\cap V|\geq 3$. Assume that $V^{\prime }=V\setminus \{v_{1}\}\subseteq N_{G^{b}}(x_{1})$. As $C_{4}⊈G^{b}$, we have $|N_{G^{r}}(w)\cap V^{\prime }|\geq 2$ for each $w\in \{y_{1},x_{2}\}$, hence as $|V^{\prime }|=3$, we have $|N_{G^{r}}(x_{2})\cap N_{G^{r}}(y_{1})\cap V^{\prime }|\geq 1$. Therefore $C_{3}\subseteq G^{r}$, a contradiction. Thus, this case is not possible.

**Case 2**: $|E(K)\cap E(G^{b})|=2$. W.l.g suppose that $x_{1}y_{1},x_{1}z_{1}\in E(G^{b})$ and $y_{1}z_{1}\in E(G^{r})$. Since $C_{4}⊈G^{b}$, there exists at least one edge between $\left\{z_{1},y_{1}\right\}$ and $\left\{x_{2},x_{3}\right\}$ in $G^{r}$. W.l.g assume that $y_{1}x_{2}\in E(G^{r})$. Since $y_{1}x_{2},y_{1}z_{1}\in E(G^{r})$, if $|N_{G^{r}}(y_{1})\cap V|\geq 2$, then $C_{4}\subseteq G^{b}[N_{G^{r}}(y_{1})]$, a contradiction. Therefore $|N_{G^{b}}(y_{1})\cap V|\geq 3$. Assume that $V^{\prime }=V\setminus \{v_{1}\}\subseteq N_{G^{b}}(y_{1})$. Since $C_{4}⊈G^{b}$, we have $|N_{G^{r}}(w)\cap V^{\prime }|\geq 2$ for each $w\in V(H)\setminus (V\cup \{y_{1}\})$. Also, there exists at least one edge between $\left\{x_{1},z_{1}\right\}$ and $\left\{y_{2},y_{3}\right\}$ in $G^{r}$. W.l.g assume that $x_{1}y_{2}\in E(G^{r})$. Hence, as $|V^{\prime }|=3$, we can say that $|N_{G^{r}}(x_{1})\cap N_{G^{r}}(y_{2})\cap V^{\prime }|\geq 1$. Therefore $C_{3}\subseteq G^{r}$, a contradiction. So, this case is not possible.

**Case 3**: $|E(K)\cap E(G^{b})|=1$. W.l.g assume that $x_{1}y_{1},x_{1}z_{1}\in E(G^{r})$ and $y_{1}z_{1}\in E(G^{b})$. If $|N_{G^{r}}(x_{1})\cap V|\geq 2$, then $C_{4}\subseteq G^{b}[N_{G^{r}}(x_{1})]$, a contradiction. Hence $|N_{G^{b}}(x_{1})\cap V|\geq 3$. Assume that $V^{\prime }=V\setminus \{v_{1}\}\subseteq N_{G^{b}}(x_{1})$. As $C_{4}⊈G^{b}$, we have $|N_{G^{r}}(w)\cap V^{\prime }|\geq 2$ for each $w\in V(H)\setminus (V\cup \{x_{1}\})$. Also, there exists at least one edge between $\left\{y_{1},z_{1}\right\}$ and $\left\{x_{2},x_{3}\right\}$ in $G^{r}$, assume that $e=ww^{\prime }$ is this edge. Since $|V^{\prime }|=3$ and $|N_{G^{r}}(w)\cap V^{\prime }|\geq 2$, we can say that $|N_{G^{r}}(w)\cap N_{G^{r}}(w)\cap V^{\prime }|\geq 1$. Therefore, $C_{3}\subseteq G^{r}$, a contradiction. So this case is not possible.

Hence by Cases 1, 2, 3, the assumption does not hold, and the theorem holds. □

Lemma 3.5 *For each red-blue coloring of the edges of*$H=K_{2,4}⊕P_{3}$*say*$\left(G^{r},G^{b}\right)$*, either*$C_{3}\subseteq G^{r}$*or*$C_{4}\subseteq G^{b}$*.*

Proof For i∈[3], suppose that $X_{i}$ is a partition set of *H*, where $X_{1}=\{x_{1}^{1},x_{2}^{1},x_{3}^{1}\}$, $X_{2}=\{x_{1}^{2},x_{2}^{2}\}$, and $X_{3}=\{x_{1}^{3},\ldots ,x_{4}^{3}\}$. Also assume that $P_{3}=x_{1}^{1}x_{2}^{1}x_{3}^{1}$. By contrary, suppose that $C_{3}⊈G^{r}$ and $C_{4}⊈G^{b}$. Therefore, as $C_{4}⊈G^{b}$, we have $|N_{G^{r}}(v)|\leq 4$ for each $v\in X_{3}$. Otherwise $C_{4}\subseteq G^{b}[N_{G^{r}}(v)]$. Since $|X_{3}|=4$, one can say that there are at least two members $v,v^{\prime }$ of $X_{3}$, so that $|N_{G^{r}}(v)|\geq 3$ and $|N_{G^{r}}(v^{\prime })|\geq 3$. Otherwise one can say that $C_{4}\subseteq [X_{1}\cup X_{2},X_{3}]$. W.l.g let $v=x_{1}^{3},v^{\prime }=x_{2}^{3}$. Now, by considering the edges of $P_{3}$, we have three case as follows:

**Case 1**: $E(P_{3})\subseteq E(G^{r})$. As $C_{3}⊈G^{r}$, we have $|N_{G^{r}}(x_{2}^{1})\cap (X_{2}\cup X_{3})|\leq 1$, otherwise $C_{4}\subseteq G^{b}[\{x_{1}^{1},x_{3}^{1}\},N_{G^{r}}(x_{2}^{1})]$. Therefore, $|N_{G^{b}}(x_{2}^{1})\cap (X_{2}\cup X_{3})|\geq 5$. Hence $|N_{G^{b}}(x_{2}^{1})\cap X_{2}|\geq 1$ and $|N_{G^{b}}(x_{2}^{1})\cap X_{3}|\geq 3$. W.l.g let $\left\{x_{1}^{2},x_{1}^{3},x_{2}^{3},x_{3}^{3}\}\subseteq N_{G^{b}}(x_{2}^{1}\right)$. Since $C_{4}⊈G^{b}$, $|N_{G^{r}}(x_{1}^{2})\cap X_{3}\setminus \{x_{4}^{3}\}|\geq 2$. W.l.g assume that $\left\{x_{1}^{3},x_{2}^{3}\}\subseteq N_{G^{r}}(x_{1}^{2}\right)$. If $xx_{1}^{2}\in G^{r}$ for at least one $x\in \{x_{1}^{1},x_{3}^{1}\}$, then $C_{4}\subseteq G^{b}[\{x,x_{1}^{2}\},\{x_{1}^{3},x_{2}^{3}\}]$, a contradiction. Therefore, $X_{1}\subseteq N_{G^{b}}(x_{1}^{2})$. Hence $|N_{G^{r}}(x_{2}^{2})\cap X_{1}|\geq 2$, otherwise $C_{4}\subseteq G^{b}[X_{1},X_{2}]$, a contradiction again. W.l.g suppose that $x_{1}^{1}\in N_{G^{r}}(x_{2}^{2})$. Therefore, as $C_{4}⊈G^{b}$, it can be checked that $|N_{G^{r}}(x_{i}^{i})\cap \{x_{1}^{3},x_{2}^{3},x_{3}^{3}\}|\geq 2$ for i=1,2. So, $C_{3}\subseteq G^{r}[x_{1}^{1},x_{2}^{2},x]$ for some $x\in \{x_{1}^{3},x_{2}^{3},x_{3}^{3}\}$.

**Case 2**: $E(P_{3})\subseteq E(G^{b})$. Since $C_{4}⊈G^{b}$, $|N_{G^{r}}(x)\cap \{x_{1}^{1},x_{3}^{1}\}|\geq 1$ for each $x\in X_{2}\cup X_{3}$. In case there exists a vertex *α* of $X_{2}$, so that $|N_{G^{b}}(\alpha )\cap X_{3}|\geq 3$, as $C_{4}⊈G^{b}$ and $|N_{G^{r}}(x)\cap \{x_{1}^{1},x_{3}^{1}\}|\geq 1$ for each $x\in X_{2}$, then in any case $C_{3}\subseteq G^{r}[\{x^{\prime },\alpha^{\prime },x^{\prime \prime }\}]$ for some $x^{\prime }\in X_{1}$, $\alpha \neq \alpha^{\prime }\in X_{2}$, and $x^{\prime \prime }\in N_{G^{b}}(\alpha )\cap X_{3}$. Hence, assume that $|N_{G^{b}}(\alpha )\cap X_{3}|\leq 2$ for each $\alpha \in X_{2}$. If there exists a vertex *β* of $X_{2}$, such that $|N_{G^{b}}(\beta )\cap X_{3}|\leq 1$, as $C_{3}⊈G^{r}$ and $|N_{G^{r}}(x)\cap \{x_{1}^{1},x_{3}^{1}\}|\geq 1$ for each $x\in X_{2}$, there is at least one vertex of $\left\{x_{1}^{1},x_{3}^{1}\right\}$ say $\beta^{\prime }$, so that $|N_{G^{b}}(\beta^{\prime })\cap X_{3}|\geq 3$. W.l.g bear in mind that $|N_{G^{b}}(x_{1}^{2})\cap X_{3}|\leq 1$ and $\left|N_{G^{r}}(x_{1}^{2})\cap \{x_{1}^{1},x_{3}^{1}\}=\{x_{1}^{1}\right\}$. Hence $|N_{G^{b}}(x_{1}^{1})\cap X_{3}|\geq 3$. Now by considering $A=\{x_{2}^{1},x_{3}^{1},x_{2}^{2}\}$, one can say that either $C_{4}\subseteq G^{b}[\{x_{2}^{1},x_{3}^{1}\},X_{2}]$ or $C_{4}\subseteq G^{b}[\{x_{1}^{1},x\},X_{3}]$ for some x∈A, a contradiction. Therefore assume that $|N_{G^{b}}(\gamma )\cap X_{3}|=2$ for each $\gamma \in X_{2}$. W.l.g suppose that $\left\{x_{1}^{1},x_{1}^{3},x_{2}^{3}\}\subseteq N_{G^{r}}(x_{1}^{2}\right)$. Since $C_{4}⊈G^{b}$ and $C_{3}⊈G^{r}$, we have $\left\{x_{1}^{1}x_{1}^{3},x_{1}^{1}x_{2}^{3},x_{2}^{1}x_{1}^{2},x_{3}^{1}x_{1}^{2}\}\subseteq E(G^{b}\right)$ and $G[\{x_{2}^{1},x_{3}^{1}\},\{x_{3}^{3},x_{4}^{3}\}]\subseteq G^{r}$. Since $C_{4}⊈G^{b}$, we can say that $|N_{G^{r}}(x_{2}^{2})\cap \{x_{3}^{3},x_{4}^{3}\}|\geq 1$. Now as $C_{3}⊈G^{r}$, we have $C_{4}\subseteq G^{b}[\{x_{2}^{1},x_{3}^{1}\},X_{2}]$, which is a contradiction again.

**Case 3**: $|E(P_{3})\cap E(G^{b})|=1$. W.l.g suppose that $x_{1}^{1}x_{2}^{1}\in E(G^{b})$ and $x_{2}^{1}x_{3}^{1}\in E(G^{r})$. If there exists a vertex of $X_{2}$ say *v*, so that $|N_{G^{b}}(v)\cap X_{3}|\geq 3$, since $C_{4}⊈G^{b}$, we have $|N_{G^{r}}(x)\cap (N_{G^{b}}(v)\cap X_{3})|\geq 2$ for each $x\in X_{1}$. Hence it is easy to say that $C_{3}\subseteq G^{r}[\{x_{2}^{1},x_{3}^{1}\},\{v^{\prime }\}]$ for some $v^{\prime }\in N_{G^{b}}(v)\cap X_{3}$, a contradiction. Hence, assume that $|N_{G^{b}}(v)\cap X_{3}|\leq 2$ for each $v\in X_{2}$. Let there exists a vertex of $X_{2}$ say *v*, so that $|N_{G^{b}}(v)\cap X_{3}|=2$ and w.l.g suppose that $N_{G^{b}}(x_{1}^{2})\cap X_{3}=\{x_{1}^{3},x_{2}^{3}\}$. Since $C_{4}⊈G^{b}$ and $C_{3}⊈G^{r}$, we can say that $|N_{G^{b}}(x)\cap \{x_{1}^{3},x_{2}^{3}\}|\leq 1$ for each $x\in X_{1}$ and $|N_{G^{b}}(x)\cap \{x_{1}^{3},x_{2}^{3}\}|=1$ for each $x\in \{x_{2}^{1},x_{3}^{1}\}$. As $x_{2}^{1}x_{3}^{1}\in E(G^{r})$, we have $N_{G^{r}}(x_{2}^{1})\cap \{x_{1}^{3},x_{2}^{3}\}\neq N_{G^{r}}(x_{2}^{1})\cap \{x_{1}^{3},x_{2}^{3}\}$. Therefore w.l.g suppose that $x_{2}^{1}x_{1}^{3},x_{3}^{1}x_{2}^{3}\in E(G^{r})$, that is $x_{2}^{1}x_{2}^{3},x_{3}^{1}x_{1}^{3}\in E(G^{b})$. Hence, $x_{1}^{1}x_{1}^{2}\in E(G^{r})$, otherwise $C_{4}\subseteq G^{b}$, a contradiction. Therefore, as $C_{3}⊈G^{r}$, $\left\{x_{3}^{3},x_{3}^{4}\}\subseteq N_{G^{r}}(x_{1}^{2}\right)$, and $x_{1}^{1}x_{1}^{2}\in E(G^{r})$, we have $\left\{x_{3}^{3},x_{3}^{4}\}\subseteq N_{G^{b}}(x_{1}^{1}\right)$. Hence, as $x_{2}^{1}x_{3}^{1}\in E(G^{r})$, clearly $N_{G^{r}}(x_{2}^{1})\cap \{x_{3}^{3},x_{4}^{3}\}\neq N_{G^{r}}(x_{3}^{1})\cap \{x_{3}^{3},x_{4}^{3}\}$. Therefore, w.l.g let $x_{2}^{1}x_{3}^{3},x_{3}^{1}x_{4}^{3}\in E(G^{r})$, that is $x_{2}^{1}x_{4}^{3},x_{3}^{1}x_{3}^{3}\in E(G^{b})$. Hence $\left\{x_{1}^{3},x_{2}^{3}\}\subseteq N_{G^{r}}(x_{1}^{1}\right)$. So by considering $N(x_{2}^{2})$, in any case either $C_{3}\subseteq G^{r}$ or $C_{4}\subseteq G^{b}$, which is a contradiction again. Now assume that $|N_{G^{b}}(x)\cap X_{3}|\leq 1$ for each $x\in X_{2}$. Therefore w.l.g suppose that $X_{3}^{\prime }=\{x_{1}^{3},x_{2}^{3},x_{3}^{3}\}\subseteq N_{G^{r}}(x_{1}^{2})$. Now consider $x\in X_{1}$, if $xx_{1}^{2}\in E(G^{r})$, then $|N_{G^{b}}(x)\cap X_{3}^{\prime }|=3$. Since $C_{4}⊈G^{b}$, we have $|N_{G^{r}}(x^{\prime })\cap X_{3}^{\prime }|\geq 2$ for each $x^{\prime }\in X_{1}\setminus \{x\}$.

Therefore as $|N_{G^{r}}(x)\cap X_{3}|\geq 3$ for each $x\in X_{2}$, one can check that $|N_{G^{r}}(x_{2}^{2})\cap X_{3}^{\prime }|\geq 2$. Thus $C_{4}\subseteq G^{b}[X_{1}\setminus \{x\},X_{2}]$, otherwise $C_{3}\subseteq G^{r}$. □

In the following two theorems, we get the exact value of M-R-number $m_{4}(C_{3},C_{4},nK_{2})$ for small *n*.

Theorem 3.6 *Assume that*n∈[4]*. Then:*

$$m_{4}(C_{3},C_{4},nK_{2})=\left\{ \begin{array}{cc} 4\; & n=4,\\ 3\; & n=2,3,\\ 2\; & n=1. \end{array}\right.$$

**Figure 2:**
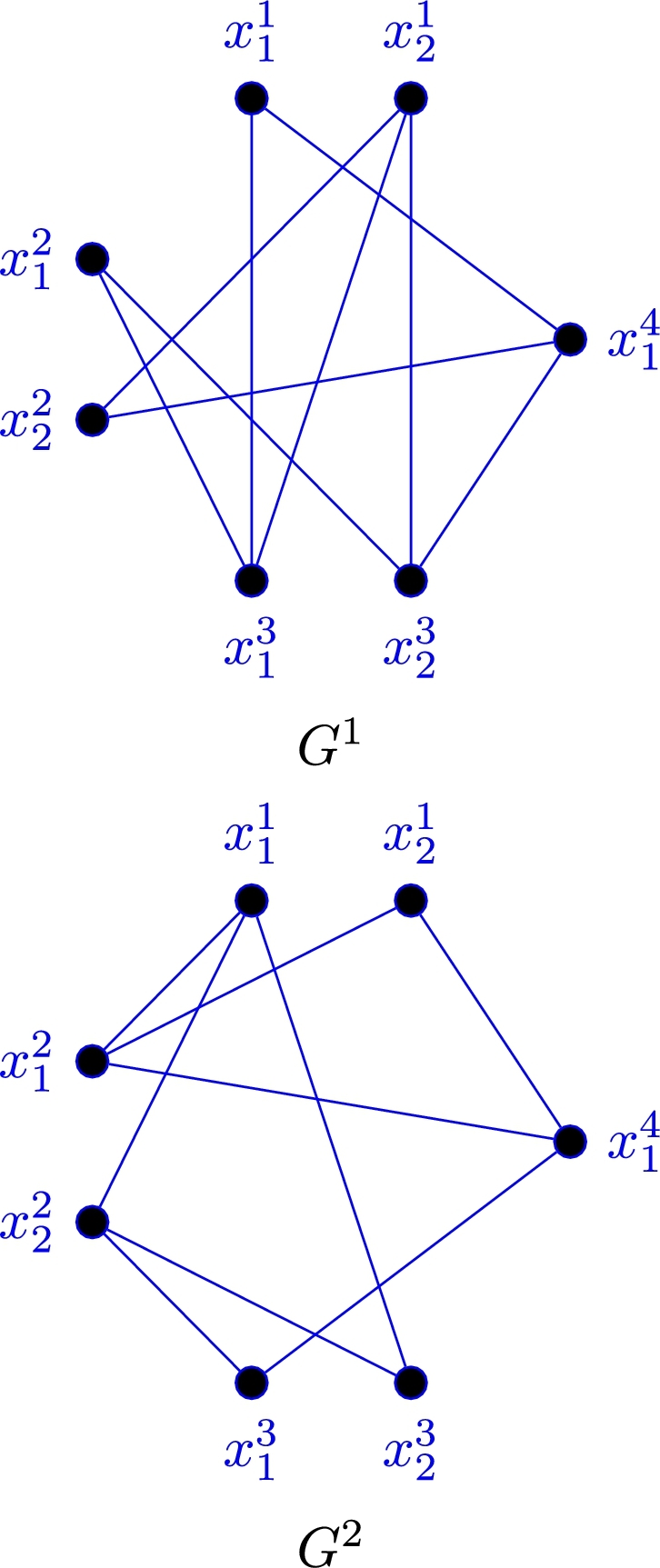
Edges decomposition of $G^{1}\cup G^{2}=\bar{G^{3}}$.

Proof For n=1, clearly $K_{4}$ is two colorable to $\left(C_{3},C_{4}\right)$, so by Theorem 3.2, $m_{4}(C_{3},C_{4},K_{2})=2$. Now assume that n=2 and consider $\left(G^{1},G^{2},G^{3}\right)$ as a 3-edge-coloring of $K=K_{4\times 2}$ with partite sets $X_{i}=\{x_{1}^{i},x_{2}^{i}\}$ for each i∈[4], where $G^{3}=K_{1,6}≅G[\{x_{2}^{4}\},X_{1}\cup X_{2}\cup X_{3}]$, and $G^{1}\cup G^{2}=\bar{G^{3}}$ as are shown in Fig. 2. Hence, it is easy to check that *K* is 3-colorable to $\left(C_{3},C_{4},2K_{2}\right)$, that is $m_{4}(C_{3},C_{4},2K_{2})\geq 3$. To prove $m_{4}(C_{3},C_{4},2K_{2})\leq 3$, contemplate a 3-edge-coloring $\left(G^{1},G^{2},G^{3}\right)$ of $K_{4\times 3}$, in which $2K_{2}⊈G^{3}$, hence $K_{4\times 2}\subseteq G^{1}\cup G^{2}$. Therefore, by Theorem 3.2 either $C_{3}\subseteq G^{1}$ or $C_{4}\subseteq G^{2}$ and the proof is complete. Now, let 3≤n≤4. Hence, we regard two cases as follows:

**Case 1**: n=3. We have $m_{4}(C_{3},C_{4},3K_{2})\geq m_{4}(C_{3},C_{4},2K_{2})=3$. Now consider $\left(G^{1},G^{2},G^{3}\right)$ as a 3-edge-coloring of $K=K_{4\times 3}$ with partite sets $X_{i}=\{x_{1}^{i},x_{2}^{i},x_{3}^{i}\}$ for each i∈[4]. W.l.g suppose that $3K_{2}⊈G^{3}$. Hence by Theorem 3.2, |M|=2, in which *M* is a M-M in $G^{3}$. W.l.g, let $M=\{e_{1},e_{2}\}$ and set $V^{\prime }=V(M)$. If $|V^{\prime }\cap X_{i}|=1$ for each i∈[4], then by Theorem 3.2 and by considering $K^{\prime }=K_{2,2,2,2}≅K\setminus \{V^{\prime }\}$, either $C_{3}\subseteq G^{1}$ or $C_{4}\subseteq G^{2}$. So assume that $|V^{\prime }\cap X_{i}|=0$ for at least one *i*, and w.l.g ponder that $|V^{\prime }\cap X_{4}|=0$. Therefore $V^{\prime }\subseteq X_{1}\cup X_{2}\cup X_{3}$ and $|V^{\prime }\cap X_{i}|=2$ for at least one i∈[3]. W.l.g suppose that $|V^{\prime }\cap X_{1}|=2$, $|V^{\prime }\cap X_{2}|=2$, and $|V^{\prime }\cap X_{3}|=0$. For other cases ($|V^{\prime }\cap X_{2}|=1$, $|V^{\prime }\cap X_{3}|=1$) the proof is identical. Now w.l.g suppose that $e_{1}=x_{1}^{1}x_{1}^{2},e_{2}=x_{2}^{1}x_{2}^{2}$. Hence $K_{1,1,3,3}≅G[\{x_{3}^{1}\},\{x_{3}^{2}\},X_{3},X_{4}]\subseteq \bar{G^{3}}$. Here by considering v∈M and *Y*, in which *Y* is the vertices of *K* that does not belong to *M*, we have Claim 3.7.

Claim 3.7 *Let*$e=v_{1}v_{2}\in E(M)$*, and w.l.g. let*$\left|N_{G^{3}}(v_{1})\cap Y|\geq |N_{G^{3}}(v_{2})\cap Y\right|$*. If*$|N_{G^{3}}(v_{1})\cap Y|\geq 2$*, then*$|N_{G^{3}}(v_{2})\cap Y|=0$*. If*$|N_{G^{3}}(v_{1})\cap Y|=1$*, then*$|N_{G^{3}}(v_{2})\cap Y|\leq 1$*and if*$|N_{G^{3}}(v_{2})\cap Y|=1$*, then*$v_{1}$*and*$v_{2}$*the same neighbor exists in Y.*

Proof By contradiction. Suppose that $\left\{v,v^{\prime }\}\subseteq Y\cap N_{G^{3}}(v_{1}\right)$ and $v^{\prime \prime }\in Y\cap N_{G^{3}}(v_{2})$, in this instance, set $M^{\prime }=(M\setminus \{v_{1},v_{2}\})\cup \{v_{1}v,v_{2}v^{\prime \prime }\}$. Obviously, $M^{\prime }$ is a matching with $|M^{\prime }|=3$, which contradicts the $3K_{2}⊈G^{3}$. If $|N_{G^{3}}(v_{i})\cap Y|=1$ and $v_{i}$ has a different neighbor, then the proof is identical. □

If there exists at least one vertex *v* of $V^{\prime }$, so that $|N_{G^{3}}(v)\cap (X_{3}\cup X_{4})|=0$, then clearly $K_{3,3,3}≅G[\{v,x_{3}^{1},x_{3}^{2}\},X_{3},X_{4}]\subseteq \bar{G^{3}}$. So by Theorem 3.2, either $C_{3}\subseteq G^{1}$ or $C_{4}\subseteq G^{2}$. Therefore by Claim 3.7, $|N_{G^{3}}(v_{i})\cap (X_{3}\cup X_{4})|=1$ for each $v\in V^{\prime }$. If for each j∈[2], $N_{G^{3}}(x_{1}^{j})\cap (X_{3}\cup X_{4})=\{x\}$, and $N_{G^{3}}(x_{2}^{j})\cap (X_{3}\cup X_{4})=\{x^{\prime }\}$, where $x,x^{\prime }$ are in different parts, then one can find a $M^{\prime }=\{e_{1}^{\prime },e_{2}^{\prime }\}$, where $e_{1}^{\prime }=x_{1}^{1}x$ and $e_{2}^{\prime }=x_{2}^{2}x^{\prime }$. Now since $\left\{x_{1}^{1},x_{2}^{2},x,x^{\prime }\right\}$ are in different parts of *K*, by considering $K^{\prime \prime }=K_{2,2,2,2}≅K\setminus V(M^{\prime })$ and by Theorem 3.2, either $C_{3}\subseteq G^{1}$ or $C_{4}\subseteq G^{2}$. Hence, suppose that $N_{G^{3}}(v)\cap X_{i}=\emptyset$ for one i∈{3,4} and each $v\in V^{\prime }$. W.l.g let $N_{G^{3}}(v)\cap X_{4}=\emptyset$, in other words $|N_{G^{3}}(v)\cap X_{3}|=1$ for each $v\in V^{\prime }$. Therefore by Claim 3.7, $N_{G^{3}}(x_{1}^{1})\cap X_{3}=N_{G^{3}}(x_{1}^{2})\cap X_{3}$ and $N_{G^{3}}(x_{2}^{1})\cap X_{3}=N_{G^{3}}(x_{2}^{2})\cap X_{3}$. Suppose that $N_{G^{3}}(x_{1}^{1})\cap X_{3}=\{z\}$ and $N_{G^{3}}(x_{2}^{1})\cap X_{3}=\{z^{\prime }\}$. If $z=z^{\prime }$, then $K_{3,3,3}≅G[X_{2},X_{3}\setminus \{z\}\cup \{x_{3}^{1}\},X_{4}]\subseteq \bar{G^{3}}$. So by Theorem 3.2, either $C_{3}\subseteq G^{1}$ or $C_{4}\subseteq G^{2}$. Therefore, assume that $z\neq z^{\prime }$ and w.l.g let $z=x_{1}^{3},z^{\prime }=x_{2}^{3}$. Hence $G^{3}≅2K_{3}$ and $\bar{G^{3}}≅3K_{1}⊕H$, where $H=G[X_{1},X_{2},X_{3}]\setminus 2K_{3}$. Therefore, the following claim is issued by us:

Claim 3.8 *For any red-blue coloring*$\left(G^{r},G^{b}\right)$*of the edges of*$\bar{G^{3}}$*, either*$C_{3}\subseteq G^{r}$*or*$C_{4}\subseteq G^{b}$*.*

Proof By contradiction, suppose that $C_{3}⊈G^{r}$ and $C_{4}⊈G^{b}$ for some 2-edges coloring $\left(G^{r},G^{b}\right)$ of $\bar{G^{3}}$. If $|N_{G^{r}}(x)|\geq 7$ for some $x\in X_{4}$, then it is clear that $C_{4}\subseteq G^{b}[N_{G^{r}}(x)]$. Hence we may assume that $|N_{G^{r}}(x)|\leq 6$ for each $x\in X_{4}$. If $|N_{G^{r}}(x)|\leq 4$ for each $x\in X_{4}$, then there exists at least two vertices $x,x^{\prime }$ of $X_{4}$, so that $|N_{G^{b}}(x)\cap N_{G^{b}}(x^{\prime })|\geq 2$, which means that $C_{4}\subseteq G^{b}$ and the proof is complete. Hence, imagine that there is at least one vertex *w* of $X_{4}$, so that $5\leq |N_{G^{r}}(w)|\leq 6$. Now, w.l.g suppose that $|N_{G^{r}}(x_{1}^{4})|=6$ (for other cases the proof is the same). Let $X^{\prime }=\{x_{3}^{1},x_{3}^{2},x_{3}^{3}\}$. If $|N_{G^{r}}(x_{1}^{4})\cap X^{\prime }|=3$, then one can say that either $C_{3}\subseteq G^{r}$ or $C_{4}\subseteq G^{b}$. Thus let $|N_{G^{r}}(x_{1}^{4})\cap X^{\prime }|\leq 2$. First assume that $|N_{G^{r}}(x_{1}^{4})\cap X^{\prime }|=2$ and w.l.g assume that $N_{G^{r}}(x_{1}^{4})\cap X^{\prime }=\{x_{3}^{1},x_{3}^{2}\}$, so $x_{3}^{1}x_{3}^{2},x_{3}^{3}x_{1}^{4}\in E(G^{b})$. If $|N_{G^{r}}(x_{1}^{4})\cap X_{i}|=3$ for one i∈{1,2}, then it can be said that $C_{4}\subseteq G^{b}[N_{G^{r}}(x_{1}^{4})]$. So suppose that $|N_{G^{r}}(x_{1}^{4})\cap X_{i}|=2$ for each i∈[n]. Hence $X_{3}\setminus \{x_{3}^{3}\}\subseteq N_{G^{r}}(x_{1}^{4})$. In this case one can say that $C_{4}\subseteq [\{x_{3}^{1},x_{3}^{2},x_{1}^{3},x_{2}^{3}\}]$. Now let $|N_{G^{r}}(x_{1}^{4})\cap X^{\prime }|=1$ and w.l.g let $N_{G^{r}}(x_{1}^{4})\cap X^{\prime }=\{x_{3}^{1}\}$. If $|N_{G^{r}}(x_{1}^{4})\cap X_{1}|=3$, then one can say that $C_{4}\subseteq G^{b}$. Hence let $|N_{G^{r}}(x_{1}^{4})\cap X_{i}|=2$ for each i∈[3]. W.l.g let $N_{G^{r}}(x_{1}^{4})\cap X_{1}=\{x_{1}^{1},x_{3}^{1}\}$. As $|N_{G^{r}}(x_{1}^{4})\cap X^{\prime }|=1$, $|X_{i}|=3$, and $|N_{G^{r}}(x_{1}^{4})|=6$ for i=2,3, we have $X_{i}\setminus \{x_{3}^{i}\}\subseteq |N_{G^{r}}(x_{1}^{4})$. Therefore as $C_{3}⊈G^{r}$, one can say that $C_{4}\subseteq G^{b}[\{x_{1}^{1},x_{3}^{1},x_{2}^{2},x_{2}^{3}\}]$, a contradiction. So suppose that $|N_{G^{r}}(x_{1}^{4})\cap X^{\prime }|=0$, that is $V^{\prime }\cup \{z,z^{\prime }\}=N_{G^{r}}(x_{1}^{4})$ (for other cases the proof is the same). Therefore, as $C_{3}⊈G^{r}$, we can say that $G[N_{G^{r}}(x_{1}^{4})]\subseteq \bar{G^{3}}$ and $N_{G^{b}}(x_{1}^{4})=\{x_{3}^{1},x_{3}^{2},x_{3}^{3}\}$. Since $C_{3}⊈G^{r}$ and $C_{4}⊈G^{b}$, we have $|N_{G^{r}}(w^{\prime })\cup \{x_{3}^{1},x_{3}^{2},x_{3}^{3}\}|=2$ for each $w^{\prime }\in X_{4}\setminus \{x_{1}^{4}\}$ and also $N_{G^{r}}(x_{2}^{4})\cap \{x_{3}^{1},x_{3}^{2},x_{3}^{3}\}=N_{G^{r}}(x_{3}^{4})\cap \{x_{3}^{1},x_{3}^{2},x_{3}^{3}\}$. Otherwise $C_{4}\subseteq G^{b}[\{x_{3}^{1},x_{3}^{2},x_{3}^{3},x_{1}^{4}\}]$, a contradiction. W.l.g assume that $N_{G^{r}}(x_{i}^{4})\cap \{x_{3}^{1},x_{3}^{2},x_{3}^{3}\}=\{x_{3}^{1},x_{3}^{2}\}$ for each i∈{2,3}. Now, consider $x_{1}^{3}$, since $x_{3}^{3}x_{j}^{4}\in E(G^{b})$ for each j=1,2,3 and $C_{4}⊈G^{b}$, we have $x_{1}^{3}x_{j}^{4}\in E(G^{r})$ for at least one j∈{2,3}. W.l.g suppose that $x_{1}^{3}x_{2}^{4}\in E(G^{r})$, since $C_{3}⊈G^{r}$ and $\left\{x_{3}^{1},x_{3}^{2}\}\subseteq N_{G^{r}}(x_{2}^{4}\right)$, we can say that $x_{1}^{3}x_{3}^{j}\in E(G^{b})$ for each j∈{1,2}. Hence $C_{4}\subseteq G^{b}[\{x_{3}^{1},x_{3}^{2},x_{1}^{3},x_{1}^{4}\}]$, a contradiction again and complete is the proof. □

Therefore by Claim 3.8, the proof of Case 1 is complete.

**Case 2**: n=4. For the lower bound consider $K=K_{4\times 3}$ and respect $X_{i}=\{x_{1}^{i},x_{2}^{i},x_{3}^{i}\}$ for each i∈[4] as the partite sets of *K*. Consider $\left(G^{1},G^{2},G^{3}\right)$ as a 3-edge-coloring of *K*, where $G^{3}≅G[X_{1},X_{2},\{x_{1}^{3}\}]≅K_{3,3,1}$ and $G_{1}\cup G^{2}≅\bar{G^{3}}$. It is easy to check that $4K_{2}⊈G^{3}$ and $\bar{G^{3}}\subseteq K_{3,7,2}$. Hence by Lemma 3.3, $\bar{G^{3}}$ is 2-colorable to $\left(C_{3},C_{4}\right)$, which means that $m_{4}(C_{3},C_{4},4K_{2})\geq 4$. Now, suppose on contrary that $K=K_{4\times 4}$ is 3-colorable to $\left(C_{3},C_{4},4K_{2}\right)$. Consider $\left(G^{1},G^{2},G^{3}\right)$ as a 3-edge-coloring of $K_{4\times 4}$, where $C_{3}⊈G^{1}$, $C_{4}⊈G^{2}$, and $4K_{2}⊈G^{3}$. Since |M|≤3, in which *M* is a M-M in $G^{3}$, w.l.g suppose that $v_{1}v_{2}\in M$, where $v_{i}\in X_{i}$ for i=1,2. Set $K^{\prime }=K_{3,3,4,4}≅K\setminus \{v,v^{\prime }\}$. Therefore, as $C_{3}⊈G^{1}$, $C_{4}⊈G^{2}$, and by Case 1, $M^{\prime }=3K_{1}\subseteq G^{3}[K^{\prime }]$, which means that $M=M^{\prime }\cup \{vv^{\prime }\}=4K_{2}\subseteq G^{3}$, a contradiction. So, $m_{4}(C_{3},C_{4},4K_{2})=4$. □

Theorem 3.9 $m_{4}(C_{3},C_{4},5K_{2})=4$*.*

Proof We have $m_{4}(C_{3},C_{4},5K_{2})\geq m_{4}(C_{3},C_{4},4K_{2})=4$. Now, by contrary assume that $K=K_{4\times 4}$ with partite sets $X_{i}$ for i=1,2,3,4, be 3-colorable to $\left(C_{3},C_{4},5K_{2}\right)$. Consider $\left(G^{1},G^{2},G^{3}\right)$ as a 3-edge-coloring of $K_{4\times 4}$, where $C_{3}⊈G^{1}$, $C_{4}⊈G^{2}$, and $5K_{2}⊈G^{3}$. Hence by Case 2 of Theorem 3.6, |M|=4, in which *M* is a M-M in $G^{3}$. Let $M=\{e_{1},e_{2},e_{3},e_{4}\}$ and set $V^{\prime }=V(M)$. In case there exist two edges, say $e=vv^{\prime }$ and $e^{\prime }=uu^{\prime }$ in *M*, so that $v,v^{\prime },u$, and $u^{\prime }$ be in different parts of *K*, then by Case 1 of Theorem 3.6 and by considering $K^{\prime }=K_{3,3,3,3}≅K\setminus \{v,v^{\prime },u,u^{\prime }\}$, one can say that $M^{\prime }=3K_{2}\subseteq G^{3}[K^{\prime }]$, which means that $M^{\prime }\cup \{e,e^{\prime }\}=5K_{2}\subseteq G^{3}$, a contradiction. Hence, imagine that there is at least one i∈[4] so that $|V^{\prime }\cap X_{i}|=0$. W.l.g let $|V^{\prime }\cap X_{4}|=0$, that is $4K_{2}\subseteq G^{3}[X_{1},X_{2},X_{3}]$. As $|X_{i}|=4$ and |M|=4, there exists four vertices of $X_{1}\cup X_{2}\cup X_{3}$, say *Y*, in order that the vertices of *Y* do not belong to *M*.

Now, consider *Y* and set $\left(|Y_{1}|,|Y_{2}|,|Y_{3}|)=(|X_{i_{1}}\cap Y|,|X_{i_{2}}\cap Y|,|X_{i_{3}}\cap Y|\right)$, in a place which $i_{j}\in [3]$. Therefore $\left(|Y_{1}|,|Y_{2}|,|Y_{3}|)\in \{(4,0,0),(3,1,0),(2,2,0),(2,1,1)\right\}$. So by considering $\left(|Y_{1}|,|Y_{2}|,|Y_{3}|\right)$, we have the following cases:

**Case 1**: $\left(|Y_{1}|,|Y_{2}|,|Y_{3}|)=(4,0,0\right)$. W.l.g suppose that $i_{1}=3$. Therefore $4K_{2}\subseteq G^{3}[X_{1},X_{2}]$ and $G[X_{3},X_{4}]\subseteq \bar{G^{3}}$. W.l.g we can ponder that $e_{i}=x_{i}^{1}x_{i}^{2}$ and also w.l.g let $\left|N_{G^{3}}(x_{i}^{1})\cap Y^{\prime }|\geq |N_{G^{3}}(x_{i}^{2})\cap Y^{\prime }\right|$ for each i∈[4], where $Y^{\prime }=X_{3}\cup X_{4}$. Therefore, in case there exists at least one i∈[4], in order that $|N_{G^{3}}(x_{i}^{j})\cap Y^{\prime }|=0$ for at least one j∈[2], then by Claim 3.7, it can be said that $K_{3\times 3}\subseteq \bar{G^{3}}$. So by Theorem 3.2, either $C_{3}\subseteq G^{1}$ or $C_{4}\subseteq G^{2}$, a contradiction. Hence, as $\left|N_{G^{3}}(x_{i}^{1})\cap Y^{\prime }|\geq |N_{G^{3}}(x_{i}^{2})\cap Y^{\prime }\right|$ and by Claim 3.7 it can be said that $|N_{G^{3}}(x_{i}^{1})\cap Y^{\prime }|=|N_{G^{3}}(x_{i}^{2})\cap Y^{\prime }|=1$ for each i∈[4]. If there exist $i^{\prime },i^{\prime \prime }\in [4]$ say $i^{\prime }=1,i^{\prime \prime }=2$, so that for each j∈[2], $N_{G^{3}}(x_{1}^{j})\cap X_{3}=\{x\}$ and $N_{G^{3}}(x_{2}^{j})\cap X_{4}=\{x^{\prime }\}$, then one can find $M^{\prime }=\{e_{1}^{\prime },e_{2}^{\prime },e_{3},e_{4}\}$, where $e_{1}^{\prime }=x_{1}^{1}x$ and $e_{2}^{\prime }=x_{2}^{2}x^{\prime }$. Hence as $\left\{x_{1}^{1},x_{2}^{2},x,x^{\prime }\right\}$ are in different parts of *K*, by Case 1 of Theorem 3.6 and by considering $K^{\prime \prime }=K_{3,3,3,3}≅K\setminus V(M^{\prime })$, it can be checked that $M^{\prime \prime }=3K_{2}\subseteq G^{3}[K^{\prime \prime }]$, which means that $M^{\prime \prime }\cup \{e_{1}^{\prime },e_{2}^{\prime }\}=5K_{2}\subseteq G^{3}$, a contradiction. Therefore, suppose that $|N_{G^{3}}(x_{i}^{1})\cap X_{r}|=|N_{G^{3}}(x_{i}^{2})\cap X_{r}|=1$ for one r∈{3,4} and for each i∈[4]. So, w.l.g assume that r=3. If there exist $i^{\prime },i^{\prime \prime }\in [4]$, so that $N_{G^{3}}(x_{i^{\prime }}^{j})\cap X_{3}=N_{G^{3}}(x_{i^{\prime \prime }}^{j})\cap X_{3}=\{x\}$ for each j∈[2], then by Claim 3.7, $K_{4,3,4}\subseteq G[\{x_{i^{\prime }}^{1},x_{i^{\prime }}^{2},x_{i^{\prime \prime }}^{1},x_{i^{\prime \prime }}^{2}\},X_{3}\setminus \{x\},X_{4}]\subseteq \bar{G^{3}}$. So by Theorem 3.2, either $C_{3}\subseteq G^{1}$ or $C_{4}\subseteq G^{2}$, a contradiction. Hence suppose that $N_{G^{3}}(x_{i^{\prime }}^{j})\cap X_{3}\neq N_{G^{3}}(x_{i^{\prime \prime }}^{j})\cap X_{3}$ for each j∈[2] and each $i^{\prime },i^{\prime \prime }\in [4]$. So, since $|N_{G^{3}}(x_{i}^{1})\cap Y^{\prime }|=|N_{G^{3}}(x_{i}^{2})\cap Y^{\prime }|=1$ for each i∈[4], w.l.g assume that $N_{G^{3}}(x_{i}^{j})\cap X_{3}=\{x_{i}^{3}\}$ for each i∈[4]. Therefore $G^{3}≅4K_{3}$ and $\bar{G^{3}}≅4K_{1}⊕H$, where $H=G[X_{1},X_{2},X_{3}]\setminus 4K_{3}$. Therefore, our claim is as follows:

Claim 3.10 *For each red-blue coloring of the edges of*$\bar{G^{3}}$*say*$\left(G^{r},G^{b}\right)$*, either*$C_{3}\subseteq G^{r}$*or*$C_{4}\subseteq G^{b}$*.*

Proof By contradiction, suppose that $C_{3}⊈G^{r}$ and $C_{4}⊈G^{b}$. As $C_{3}⊈G^{r}$, we have $|N_{G^{r}}(v)|\leq 6$ for each $v\in X_{4}$. Otherwise, one can check that $C_{3}\subseteq G^{r}$ which is a contradiction. Since $|X_{i}|=4$, we can check that $|N_{G^{b}}(v)|\geq 6$ for each $v\in X_{4}$. Now since $|X_{4}|=4$, there exist at least two vertices $v,v^{\prime }$ of $X_{4}$, so that $|N_{G^{b}}(v)\cap N_{G^{b}}(v^{\prime })|\geq 2$, which means that $C_{4}\subseteq G^{b}$ and the proof is complete. □

Therefore by Claim 3.10, the proof related to Case 1 is complete.

**Case 2**: $\left(|Y_{1}|,|Y_{2}|,|Y_{3}|)=(3,1,0\right)$. W.l.g suppose that $i_{1}=3,i_{2}=2$. Therefore $4K_{2}\subseteq G^{3}[X_{1},X_{2}\setminus \{x\},\{x^{\prime }\}]$, where $x^{\prime }\in X_{3}$. W.l.g assume that $x=x_{4}^{2}$, $x^{\prime }=x_{1}^{3}$, $e_{i}=x_{i}^{1}x_{i}^{2}$ for i=1,2,3, and $e_{4}=x_{4}^{1}x_{1}^{3}$. So $G[\{x_{4}^{2}\},X_{3}\setminus \{x_{1}^{3}\},X_{4}]\subseteq \bar{G^{3}}$, otherwise $5K_{2}\subseteq G^{3}$, a contradiction. Set $X_{3}^{\prime }=X_{3}\setminus \{x_{1}^{3}\}$ and $V^{\prime \prime }=X_{1}\setminus \{x_{4}^{1}\}\cup X_{2}\setminus \{x_{4}^{2}\}$. If there exist at least two vertices $\left\{z,z^{\prime }\right\}$ of $V^{\prime \prime }$, so that $|N_{G^{3}}(v)\cap X_{3}^{\prime }|=0$ for each $v\in \{z,z^{\prime }\}$, then $K_{3,3,4}\subseteq G[\{z,z,x_{4}^{1}\},X_{3}^{\prime },X_{4}]\subseteq \bar{G^{3}}$, so by Theorem 3.2, either $C_{3}\subseteq G^{1}$ or $C_{4}\subseteq G^{2}$, a contradiction. Hence by Claim 3.7, $|N_{G^{3}}(x_{i}^{1})\cap X_{3}^{\prime }|=|N_{G^{3}}(x_{i}^{2})\cap X_{3}^{\prime }|=1$ for at least two i∈[3]. W.l.g for i=1,2, $|N_{G^{3}}(x_{i}^{1})\cap X_{3}^{\prime }|=|N_{G^{3}}(x_{i}^{2})\cap X_{3}^{\prime }|=1$. If $N_{G^{3}}(x_{i}^{1})\cap X_{3}^{\prime }=N_{G^{3}}(x_{i}^{2})\cap X_{3}^{\prime }$, then $P_{3}⊕K_{2,4}\subseteq G[\{x_{1}^{1},x_{2}^{1},x_{4}^{2}\},X_{3}^{\prime }\setminus \{x\},X_{4}]\subseteq \bar{G^{3}}$, where $\{x\}=N_{G^{3}}(x_{i}^{1})\cap X_{3}^{\prime }=N_{G^{3}}(x_{i}^{2})\cap X_{3}^{\prime }$, which is a contradiction by Lemma 3.5. Hence, w.l.g assume that $N_{G^{3}}(x_{i}^{1})\cap X_{3}^{\prime }=\{x_{2}^{3}\}$ and $N_{G^{3}}(x_{i}^{2})\cap X_{3}^{\prime }=\{x_{3}^{3}\}$ for i=1,2. Now, by considering $N_{G^{3}}(x_{3}^{1})\cap X_{3}^{\prime }$ and by Claim 3.7, we have $N_{G^{3}}(x_{3}^{j})\cap X_{3}^{\prime }=\{x_{4}^{3}\}$, otherwise in any case one can find a copy of $P_{3}⊕K_{2,4}\subseteq G[V^{\prime \prime },X_{3}^{\prime }\setminus \{x_{1}^{1}\},X_{4}]\subseteq \bar{G^{3}}$, a contradiction again. Now by considering $M^{\prime }=\{x_{1}^{1}x_{2}^{3},x_{2}^{1}x_{3}^{3},x_{3}^{1}x_{4}^{3},x_{4}^{1}x_{1}^{3}\}$ and using Case 1, the proof is complete.

**Case 3**: $\left(|Y_{1}|,|Y_{2}|,|Y_{3}|)=(2,2,0)\right\}$. W.l.g suppose that $i_{1}=3,i_{2}=2$, and assume that $e_{1}=x_{1}^{1}x_{1}^{2}$, $e_{2}=x_{2}^{1}x_{2}^{2}$, $e_{3}=x_{3}^{1}x_{1}^{3}$, and $e_{4}=x_{4}^{1}x_{2}^{3}$. Therefore, $K_{2,2,4}≅G[X_{2}^{\prime },X_{3}^{\prime },X_{4}]\subseteq \bar{G^{3}}$, where $X_{i}^{\prime }=\{x_{3}^{i},x_{4}^{i}\}$ for each i=2,3. Hence, $G[\{x_{1}^{1},x_{2}^{1}\},X_{3}^{\prime }]\subseteq \bar{G^{3}}$ and $G[\{x_{3}^{1},x_{4}^{1}\},X_{2}^{\prime }]\subseteq \bar{G^{3}}$, otherwise the proof is same as Case 2. There is at least one edge of $G[\{x_{1}^{1},x_{2}^{1}\},X_{2}^{\prime }]$ and $G[\{x_{3}^{1},x_{4}^{1}\},X_{3}^{\prime }]$ in $G^{3}$, otherwise $K_{2,4}⊕P_{3}\subseteq \bar{G^{3}}$, which is a contradiction. W.l.g suppose that $x_{1}^{1}x_{3}^{2},x_{3}^{1}x_{3}^{3}\in E(G^{3})$. Therefore, $G[X_{2}\setminus \{x_{2}^{2}\},X_{3}\setminus \{x_{2}^{3}\}]≅K_{3,3}\subseteq \bar{G^{3}}$. Hence $G[X_{2}\setminus \{x_{2}^{2}\},X_{3}\setminus \{x_{2}^{3}\},X_{4}]≅K_{3,3,4}\subseteq \bar{G^{3}}$ and by Theorem 3.2 either $C_{3}\subseteq G^{1}$ or $C_{4}\subseteq G^{2}$, a contradiction.

**Case 4**: $\left(|Y_{1}|,|Y_{2}|,|Y_{3}|)=(2,1,1)\right\}$. W.l.g suppose that $i_{1}=3$ and let $e_{1}=x_{1}^{1}x_{1}^{2}$, $e_{2}=x_{2}^{1}x_{2}^{2}$, $e_{3}=x_{3}^{1}x_{1}^{3}$, and $e_{4}=x_{3}^{2}x_{2}^{3}$. Therefore $K_{1,1,2,4}≅G[\{x_{4}^{1}\},\{x_{4}^{2}\},X_{3}^{\prime },X_{4}]\subseteq \bar{G^{3}}$, where $X_{3}^{\prime }=\{x_{3}^{3},x_{4}^{3}\}$. Set $X_{1}^{\prime }=\{x_{1}^{1},x_{2}^{1}\}$ and $X_{2}^{\prime }=\{x_{1}^{2},x_{2}^{2}\}$. So, $G[X_{1}^{\prime },\{x_{4}^{2}\}]\subseteq \bar{G^{3}}$ and $G[X_{2}^{\prime },\{x_{4}^{1}\}]\subseteq \bar{G^{3}}$. Otherwise, w.l.g assume that $x_{1}^{1}x_{4}^{2}\in G^{3}$ (for other cases the proof is the same). Therefore, $P_{3}⊕K_{2,4}≅G[\{x_{1}^{4},x_{1}^{2},x_{4}^{2}\}]⊕G[X_{3}^{\prime },X_{4}]\subseteq \bar{G^{3}}$ and by Lemma 3.5, either $C_{3}\subseteq G^{1}$ or $C_{4}\subseteq G^{2}$. If there exists at very least one vertex of $X_{1}^{\prime }\cup X_{2}^{\prime }$ say *v*, in a way that $|N_{G^{3}}(v)\cap X_{3}^{\prime }|=0$, then we can find a copy of $P_{3}⊕K_{2,4}$ in $\bar{G^{3}}$ and the proof is complete by Lemma 3.5. Hence by Claim 3.7, assume that $|N_{G^{3}}(v)\cap X_{3}^{\prime }|=1$ for each $v\in X_{1}^{\prime }\cup X_{2}^{\prime }$. Therefore, by Claim 3.7, $N_{G^{3}}(x_{i}^{1})\cap X_{3}^{\prime }=N_{G^{3}}(x_{i}^{2})\cap X_{3}^{\prime }$ for each i∈{1,2}. If $N_{G^{3}}(x_{1}^{1})\cap X_{3}^{\prime }\neq N_{G^{3}}(x_{2}^{1})\cap X_{3}^{\prime }$, then we can find a $M^{\prime }=4K_{2}$ in $G^{3}$, so that $\left(|Y_{1}|,|Y_{2}|,|Y_{3}|)=(3,1,0\right)$ for $M^{\prime }$ and the proof is complete by Case 2. Hence, w.l.g suppose that $N_{G^{3}}(v)\cap X_{3}^{\prime }=\{x_{3}^{3}\}$ for each $v\in X_{1}^{\prime }\cup X_{2}^{\prime }$. If $x_{2}^{3}x_{4}^{2}\in E(G^{3})$, then $x_{3}^{2}x_{3}^{3},x_{3}^{2}x_{4}^{3}\in E(\bar{G^{3}})$. Otherwise $5K_{2}\subseteq G^{3}$, a contradiction. Therefore, $P_{3}⊕K_{2,4}≅G[\{x_{1}^{4},x_{3}^{2},x_{4}^{2}\}]⊕G[X_{3}^{\prime },X_{4}]\subseteq \bar{G^{3}}$ and by Lemma 3.5, either $C_{3}\subseteq G^{1}$ or $C_{4}\subseteq G^{2}$. Hence assume that $x_{2}^{3}x_{4}^{2}\in E(\bar{G^{3}})$. Thus we consider the following claim:

Claim 3.11 *There exists a copy of*$H=K_{3\times 3}\setminus K_{2,2,2}$*in*$\bar{G^{3}}$*.*

Proof Set $X_{1}^{\prime \prime }=X_{1}\setminus \{x_{3}^{1}\}$, $X_{2}^{\prime \prime }=X_{2}\setminus \{x_{3}^{2}\}$, and $X_{3}^{\prime \prime }=X_{3}\setminus \{x_{1}^{3}\}$, therefore $G[X_{1}^{\prime \prime },X_{2}^{\prime \prime },X_{3}^{\prime \prime }]≅K_{3\times 3}\setminus K_{3\times 2}\subseteq \bar{G^{3}}$. □

Now, since $H\subseteq G^{1}\cup G^{2}$ and $V(M)\cap X_{4}=\emptyset$, by Claim 3.11, $H^{\prime }=4K_{1}⊕H≅G[V(H),X_{4}]\subseteq G^{1}\cup G^{2}$. Now by Lemma 3.4, the proof of this case is complete. Thus, we can conclude that the proof of the theorem is complete by Cases 1-4. □

In the below-mentioned theorem, for each n≥1 we determine the exact value of the size of $m_{4}(C_{3},C_{4},nK_{2})$.

Theorem 3.12 *Assume that n is a positive integer, where*n≥1*and*n=3k+r*, in which*k≥1,r∈{0,1,2}*, then:*

$$m_{4}(C_{3},C_{4},nK_{2})=\left\{ \begin{array}{cc} \lceil \frac{2n}{3}\rceil +1\;\;\;\;\;\;\; & r=0,1,\\ \lceil \frac{2n}{3}\rceil \;\;\;\;\;\;\;\;\;\; & r=2. \end{array}\right.$$

Proof For lower bound, suppose that r∈{0,1}, consider $K=K_{4\times t}$ and respect $X_{i}=\{x_{1}^{i},x_{2}^{i},\ldots ,x_{t}^{i}\}$ for each i∈[4] as the partite sets of *K*, where $t=\lceil \frac{2n}{3}\rceil$. Consider $\left(G^{1},G^{2},G^{3}\right)$ as a 3-edge-coloring of *K*, where $G^{3}≅G[X_{i_{1}},X_{i_{2}},X_{i_{3}}\setminus \{v,v^{\prime }\}]≅K_{t,t,t-2}$ for some $i_{j}\in [4]$ and $G^{1}\cup G^{2}≅\bar{G^{3}}$. By definition $G^{i}$ and by Lemma 3.3, it can be seen that $G^{1}\cup G^{2}≅\bar{G^{3}}$ is 2-colorable to $\left(C_{3},C_{4}\right)$. Also by Theorem 3.1, $|M|=\lfloor \frac{3t-2}{2}\rfloor =\lfloor \frac{3t}{2}\rfloor -1\leq 2(\frac{n-1}{2})=n-1$, where *M* is a M-M in $G^{3}$. Hence, $nK_{2}⊈G^{3}$. Since $K=G^{1}\cup G^{2}\cup G^{3}$, we have $K_{4\times t}$ is 3-colorable to $\left(C_{3},C_{4},nK_{2}\right)$, that is $m_{4}(C_{3},C_{4},nK_{2})\geq t+1$, where r=0,1 and $t=\lceil \frac{2n}{3}\rceil$. Now, assume that r=2 and consider $K=K_{4\times t}$ alongside partite sets $X_{i}=\{x_{1}^{i},x_{2}^{i},\ldots ,x_{t}^{i}\}$ for each i∈[4], in which $t=\lceil \frac{2n}{3}\rceil -1$. Consider $\left(G^{1},G^{2},G^{3}\right)$ as a 3-edge-coloring of *K*, $G^{3}≅G[X_{i_{1}},\ldots X_{i_{2}},X_{i_{3}}\setminus \{v,v^{\prime }\}]≅K_{t,t,t-2}$ for some $i_{j}\in [4]$ and $G_{1}\cup G^{2}≅\bar{G^{3}}$, then the proof is the same as the case that r∈{0,1}. Which means that the lower bound holds.

By induction on *n*, we prove the upper bounds. For n=1,2,…,5, the theorem holds by Theorem 3.6, Theorem 3.9. Keep in mind that for any $n^{\prime }\leq n-1$, the theorem holds. Now, consider three cases as follows:

**Case 1:**n=3k, where k≥2. By contrary suppose that $K_{4\times t}$ with partite sets $X_{i}=\{x_{1}^{i},x_{2}^{i},\ldots ,x_{t}^{i}\}$ for i=1,2,3,4, in which $t=\lceil \frac{2n}{3}\rceil +1$, is 3-colorable to $\left(C_{3},C_{4},nK_{2}\right)$, that is there exists a 3-edge-coloring $\left(G^{1},G^{2},G^{3}\right)$ of $K_{4\times t}$, where $C_{3}⊈G^{1}$, $C_{4}⊈G^{2}$, and $nK_{2}⊈G^{3}$. W.l.g let $e=x_{1}^{1}x_{1}^{2}\in E(G^{3})$. Now, set $X_{i}^{\prime }=X_{i}\setminus \{x_{1}^{i}\}$ for each i∈[4], and contemplate $K^{\prime }=K_{4\times (t-1)}=G[X_{1}^{\prime },\ldots ,X_{4}^{\prime }]$. Take $\left(G^{\prime \;1},G^{\prime \;2},G^{\prime \;3}\right)$ as a 3-edge-coloring of $K^{\prime }$. As $m_{4}(C_{3},C_{4},(n-1)K_{2})=m_{4}(C_{3},C_{4},(3(k-1)+2)K_{2})\leq \lceil \frac{2(n-1)}{3}\rceil =2k$, $C_{3}⊈G^{\prime \;1}\subseteq G^{1}$, and $C_{4}⊈G^{\prime \;2}\subseteq G^{2}$, one can say that $(n-1)K_{2}\subseteq G^{\prime \;3}\subseteq G^{3}$. Let $M^{\prime }$ is a M-M in $G^{\prime \;3}$. Consequently, $M=M^{\prime }\cup \{e\}$ is a matching of size *n* in $G^{3}$, which is a contradiction. That means $m_{4}(C_{3},C_{4},nK_{2})\leq 2k+1$, where n=3k and k≥2.

**Case 2:**n=3k+1. The proof is identical to Case 1.

**Case 3:**n=3k+2, where k≥2. By contradiction, suppose that $K_{4\times t}$ with partite sets $X_{i}=\{x_{1}^{i},x_{2}^{i},\ldots ,x_{t}^{i}\}$ for i=1,2,3,4, in which $t=\lceil \frac{2n}{3}\rceil$ is 3-colorable to $\left(C_{3},C_{4},nK_{2}\right)$, which is a 3-edge-coloring $\left(G^{1},G^{2},G^{3}\right)$ of $K_{j\times t}$ exists, where $C_{3}⊈G^{1}$, $C_{4}⊈G^{2}$, and $nK_{2}⊈G^{3}$. Suppose that *M* is a M-M in $G^{3}$, by Cases 1, 2 it can be seen that |M|=n−1. Now, our claim is as follows:

Claim 3.13 *If there exist two edges, say*$e_{1}=uv$*and*$e_{2}=u^{\prime }v^{\prime }$*in*E(M)*, so that*$v,v^{\prime },u$*, and*$u^{\prime }$*be in different parts, then*$nK_{2}\subseteq G^{3}$*.*

Proof W.l.g. suppose that $e_{1}=x_{1}^{1}x_{1}^{2}$ and $e_{2}=x_{1}^{3}x_{1}^{4}$ are two edges in $E(M)=E((n-1)K_{2})$. Now, set $X_{i}^{\prime }=X_{i}\setminus \{x_{1}^{i}\}$ for each i∈[4], and consider $\left(G^{\prime \;1},G^{\prime \;2},G^{\prime \;3}\right)$ as a 3-edge-coloring of $K^{\prime }=K_{4\times (t-1)}=G[X_{1}^{\prime },\ldots ,X_{1}^{\prime }]$. Since $m_{4}(C_{3},C_{4},(n-2)K_{2})=m_{4}(C_{3},C_{4},3K_{2})\leq t-1$, $C_{3}⊈G^{\prime \;1}\subseteq G^{1}$, $C_{4}⊈G^{\prime \;2}\subseteq G^{2}$, we can say that $(n-2)K_{2}\subseteq G^{\prime \;3}\subseteq G^{3}$. Suppose that $M^{\prime }$ is a M-M in $G^{\prime \;3}$. Subsequently, $M=M^{\prime }\cup \{e_{1},e_{2}\}$ is a matching of size *n* in $G^{3}$, which is a conflict. □

Therefore, since $nK_{2}⊈G^{3}$ and by Claim 3.13, $|E(M)\cap G[X_{i},X_{i^{\prime }}]|=0$ for at least one $\left(i,i^{\prime }\right)$, where $i,i^{\prime },\in [4]$. W.l.g suppose that $|E(M)\cap G[X_{3},X_{4}]|=0$, that is $G[X_{3},X_{4}]\subseteq \bar{G^{3}}$. Since |V(M)|=2(n−1) and $|X_{i}|=t$, at least one i∈[4] exists so that $|E(M)\cap G[X_{i},X_{t}]|\neq 0$ for at least two t∈{1,2,3,4}≠i. W.l.g let $|E(M)\cap G[X_{1},X_{2}]|\neq 0$, $|E(M)\cap G[X_{1},X_{3}]|\neq 0$, and $e_{1}=x_{1}^{1}x_{1}^{2}$, $e_{2}=x_{2}^{1}x_{1}^{3}$ be two edges in $E(M)=E((n-1)K_{2})$. Hence by Claim 3.13, $|E(M)\cap G[X_{i},X_{4}]|=0$ for each i∈{2,3}. As k≥2, if $|E(M)\cap G[X_{2},X_{3}]|=0$, then clearly $K_{3,3,3}\subseteq G[X_{2},X_{3},X_{4}]\subseteq \bar{G^{3}}$. So by Theorem 3.2, either $C_{3}\subseteq G^{1}$ or $C_{4}\subseteq G^{2}$, a contradiction. Hence $|E(M)\cap G[X_{2},X_{3}]|\neq 0$ and w.l.g suppose that $e_{3}=x_{2}^{2}x_{2}^{3}$ be an edge in $E(M)=E((n-1)K_{2})$, therefore by Claim 3.11, $|E(M)\cap G[X_{1},X_{4}]|=0$. So $G[X_{i},X_{4}]\subseteq \bar{G^{3}}$ for each i∈[3]. Since |M|=n−1 and $V(M)\subseteq X_{1}\cup X_{2}\cup X_{3}$, there exists four vertices of $X_{1}\cup X_{2}\cup X_{3}$, say $Y=\{v_{1},\ldots ,v_{4}\}$, in order that the vertices of *Y* would not belong to *M*. Now, consider *Y* and set $\left(|Y_{1}|,|Y_{2}|,|Y_{3}|)=(|X_{i_{1}}\cap Y|,|X_{i_{1}}\cap Y|,|X_{i_{1}}\cap Y|\right)$, where $i_{j}\in [3]$. Therefore $\left(|Y_{1}|,|Y_{2}|,|Y_{3}|)\in \{(4,0,0),(2,2,0),(3,1,0),(2,1,1)\right\}$. As n=3k+2,k≥2, and t≥6, |M|=n−1, and |V(M)|=2n−2, it is straightforward to check that $|E(M)\cap E(G[X_{i},X_{t}])|\geq 4$ for each i,t∈[3]. Hence by considering $\left(|Y_{1}|,|Y_{2}|,|Y_{3}|\right)$ the cases are as follows:

**Case 3-1**: $\left(|Y_{1}|,|Y_{2}|,|Y_{3}|)=(4,0,0\right)$. W.l.g surmise that $|Y_{1}\cap X_{3}|\geq 4$, therefore $|E(M)\cap G[X_{1},X_{2}]|\geq 4$. Otherwise, as k≥2, we can see that |M|≤n−2, a contradiction. Hence assume that $|E(M)\cap G[X_{1}^{\prime },X_{2}^{\prime }]|=4$, where $|X_{i}^{\prime }|=4$ and $X_{i}^{\prime }\subseteq X_{i}$ for each i∈[2]. Hence, as $G[X_{i},X_{4}]\subseteq \bar{G^{3}}$ for each i∈[3] and by Theorem 3.9, either $C_{3}\subseteq G^{1}[X_{1}^{\prime },X_{2}^{\prime },Y,X_{4}]$ or $C_{4}\subseteq G^{2}[X_{1}^{\prime },X_{2}^{\prime },Y,X_{4}]$, a contradiction.

**Case 3-2**: $\left(|Y_{1}|,|Y_{2}|,|Y_{3}|)=(3,1,0\right)$. W.l.g suppose that $|Y_{1}\cap X_{3}|=3,|Y_{1}\cap X_{2}|=1$, therefore $|E(M)\cap G[X_{1},X_{2}]|\geq 3$ and $|E(M)\cap G[X_{1},X_{3}]|\geq 1$, otherwise as k≥2, it can be said that |M|≤n−2, a contradiction. Hence assume that $|E(M)\cap G[X_{1}^{\prime },X_{2}^{\prime }]|=3$, where $|X_{i}^{\prime }|=3$ and $X_{i}^{\prime }\subseteq X_{i}$ for each i∈[2], $vv^{\prime }\in E(M)\cap G[X_{1},X_{2}]$, where $v\in X_{1}\setminus X_{1}^{\prime }$ and $v^{\prime }\in X_{3}\setminus Y$. Hence, as $G[X_{i},X_{4}]\subseteq \bar{G^{3}}$ for each i∈[3] and by Theorem 3.9, either $C_{3}\subseteq G^{1}[X_{1}^{\prime }\cup \{v\},X_{2}^{\prime }\cup (Y\cap X_{2}),(Y\cap X_{3})\cup \{v^{\prime }\},X_{4}]$ or $C_{4}\subseteq G^{2}[X_{1}^{\prime }\cup \{v\},X_{2}^{\prime }\cup (Y\cap X_{2}),(Y\cap X_{3})\cup \{v^{\prime }\},X_{4}]$, a contradiction.

**Case 3-3**: $\left(|Y_{1}|,|Y_{2}|,|Y_{3}|)=(2,2,0\right)$. W.l.g surmise that $|Y\cap X_{3}|=2,|Y\cap X_{2}|=2$, therefore for each i=2,3, $|E(M)\cap G[X_{1},X_{i}]|\geq 2$, otherwise as k≥2, it can be said that |M|≤n−2, a contradiction. Hence assume that $|E(M)\cap G[X_{1}^{\prime },X_{2}^{\prime }]|=2$ and $|E(M)\cap G[X_{1}^{\prime \prime },X_{3}^{\prime }]|=2$, where $|X_{i}^{\prime }|=|X_{i}^{\prime \prime }|=2$, $X_{i}^{\prime }\subseteq X_{i}\setminus Y$ for each i∈{2,3}, and $X_{1}^{\prime },X_{1}^{\prime \prime }\subseteq X_{1}$. Hence, as $G[X_{i},X_{4}]\subseteq \bar{G^{3}}$ for each i∈[3] and by Theorem 3.9, either $C_{3}\subseteq G^{1}[(X_{1}^{\prime }\cup X_{1}^{\prime \prime }),X_{2}^{\prime }\cup (Y\cap X_{2}),X_{3}^{\prime }\cup (Y\cap X_{3}),X_{4}]$ or $C_{4}\subseteq G^{2}[(X_{1}^{\prime },X_{1}^{\prime \prime }),X_{2}^{\prime }\cup (Y\cap X_{2}),X_{3}^{\prime }\cup (Y\cap X_{3}),X_{4}]$, a contradiction.

**Case 3-4**: $\left(|Y_{1}|,|Y_{2}|,|Y_{3}|)=(2,1,1\right)$. W.l.g suppose that $|Y\cap X_{3}|=2,|Y\cap X_{i}|=1$ for i=1,2. Therefore for each i=1,2,3, one can check that $|E(M)\cap G[X_{i},X_{j}]|\geq 2$, otherwise as k≥2, it can be said that |M|≤n−2, a contradiction. Hence the proof is identical to Case 3-3.

Therefore by Cases 3-1, 3-2, 3-3, and 3-4 the Case 3 holds, and by Cases 1-3 the proof is complete. □

## 4 Discussion

In this paper, as the first goal we proved that if $j,i,n_{1},\ldots n_{i}$ are i+2 positive integers, where 2≤j≤5, and $i,n_{i}\geq 1$, then $m_{j}(C_{3},C_{3},n_{1}K_{2},n_{2}K_{2},\ldots ,n_{i}K_{2})=\infty$. For 6≤j≤10, then we provided a lower bound for $m_{j}(C_{3},C_{3},n_{1}K_{2},vn_{2}K_{2},\ldots ,n_{i}K_{2})$, which states that $1+\lfloor \frac{\sum_{k=1}^{k=i}(n_{k}-1)}{j-5}\rfloor \leq m_{j}(C_{3},C_{3},n_{1}K_{2},n_{2}K_{2},\ldots ,n_{i}K_{2})$. Also, for j≥6 we obtained the upper bound $\lfloor \frac{2\sum_{k=1}^{k=i}(n_{k}-1)}{j-5}\rfloor +1$ for $m_{j}(C_{3},C_{3},n_{1}K_{2},n_{2}K_{2},\ldots ,n_{i}K_{2})$. To approach above results, we used a theorem in classical Ramsey number which states that $R(C_{3},C_{3})=6$.

As the second goal, we computed the exact value of $m_{4}(C_{3},C_{4},nK_{2})$ for every positive integer *n*. Indeed we proved that $m_{4}(C_{3},C_{4},nK_{2})=\left\{ \begin{array}{cc} \lceil \frac{2n}{3}\rceil \; & n=3k+2,\;k\geq 1,\\ \lceil \frac{2n}{3}\rceil +1\; & otherwise. \end{array}\right.$

To approach the proof of the second goal, we first obtained the accurate value of M-R-number $m_{4}(C_{3},C_{4},nK_{2})$ for n∈{1,2,3,4,5} in two theorems separately, then by using these two theorems, we proved our goal.

In the rest of this segment, we put forward two problems regarding the concepts of the current paper. The first problem is related to $m_{j}(C_{3},C_{3},nK_{2},mK_{2})$, which we express as follows:

Problem 4.0.1 Suppose that j≥7 and n≥m≥2. Considering $m_{j}(C_{3},C_{3},(n-1)K_{2},mK_{2})=\lceil \frac{2n+m-3}{3}\rceil$, it follows that:

$$m_{j}(C_{3},C_{3},nK_{2},mK_{2})=\lceil \frac{2n+m-1}{3}\rceil .$$

The second problem is related to $m_{j}(C_{3},C_{4},nK_{2})$, which we express as follows:

Problem 4.0.2 Suppose that *n* be a positive integer, then:

$$m_{j}(C_{3},C_{4},nK_{2})=\left\{ \begin{array}{cc} \lceil \frac{2n}{j-1}\rceil ;\; & n=(j-1)k+r,\;k,r\geq 1,\\ \lceil \frac{2n}{j-1}\rceil +1;\; & otherwise. \end{array}\right.$$

## Declarations

### Author contribution statement

Yaser Rowshan: Conceived and designed the experiments; Performed the experiments; Analyzed and interpreted the data; Contributed reagents, materials, analysis tools or data; Wrote the paper.

Mostafa Gholami: Conceived and designed the experiments; Performed the experiments; Analyzed and interpreted the data; Wrote the paper.

Stanford Shateyi: Analyzed and interpreted the data; Wrote the paper.

### Funding statement

This research did not receive any specific grant from funding agencies in the public, commercial, or not-for-profit sectors.

### Data availability statement

No data was used for the research described in the article.

### Declaration of interests statement

The authors declare no conflict of interest.

### Additional information

No additional information is available for this paper.
